# A systematic review on the effects of exercise on gut microbial diversity, taxonomic composition, and microbial metabolites: identifying research gaps and future directions

**DOI:** 10.3389/fphys.2023.1292673

**Published:** 2023-12-19

**Authors:** John M. A. Cullen, Shahim Shahzad, Jaapna Dhillon

**Affiliations:** Department of Nutrition and Exercise Physiology, University of Missouri, Columbia, MO, United States

**Keywords:** gut microbiome, SCFA, aerobic, resistance training, HIIT, metabolic health, exercise, training

## Abstract

The gut microbiome, hosting a diverse microbial community, plays a pivotal role in metabolism, immunity, and digestion. While the potential of exercise to influence this microbiome has been increasingly recognized, findings remain incongruous. This systematic review examined the effects of exercise on the gut microbiome of human and animal models. Databases (i.e., PubMed, Cochrane Library, Scopus, and Web of Science) were searched up to June 2022. Thirty-two exercise studies, i.e., 19 human studies, and 13 animal studies with a minimum of two groups that discussed microbiome outcomes, such as diversity, taxonomic composition, or microbial metabolites, over the intervention period, were included in the systematic review (PROSPERO registration numbers for human review: CRD42023394223). Results indicated that over 50% of studies found no significant exercise effect on human microbial diversity. When evident, exercise often augmented the Shannon index, reflecting enhanced microbial richness and evenness, irrespective of disease status. Changes in beta-diversity metrics were also documented with exercise but without clear directionality. A larger percentage of animal studies demonstrated shifts in diversity compared to human studies, but without any distinct patterns, mainly due to the varied effects of predominantly aerobic exercise on diversity metrics. In terms of taxonomic composition, in humans, exercise usually led to a decrease in the Firmicutes/Bacteroidetes ratio, and consistent increases with *Bacteroides* and *Roseburia* genera. In animal models, *Coprococcus*, another short chain fatty acid (SCFA) producer, consistently rose with exercise. Generally, SCFA producers were found to increase with exercise in animal models. With regard to metabolites, SCFAs emerged as the most frequently measured metabolite. However, due to limited human and animal studies examining exercise effects on microbial-produced metabolites, including SCFAs, clear patterns did not emerge. The overall risk of bias was deemed neutral. In conclusion, this comprehensive systematic review underscores that exercise can potentially impact the gut microbiome with indications of changes in taxonomic composition. The significant variability in study designs and intervention protocols demands more standardized methodologies and robust statistical models. A nuanced understanding of the exercise-microbiome relationship could guide individualized exercise programs to optimize health.

**Systematic Review Registration:** https://www.crd.york.ac.uk/prospero/display_record.php?RecordID=394223, identifier CRD42023394223.

## 1 Introduction

The gut microbiome is a complex and diverse ecosystem of microorganisms that plays a vital role in various physiological processes, including digestion, immunity, and metabolism (Thursby and Juge, 2017). In recent years, the potential of exercise to modulate the gut microbiome has gained considerable attention, and numerous studies have investigated the effects of exercise on the gut microbiome.

The first indications that exercise has a role in modulating the human gut microbiome and metabolic capacity of the microbiota were observed through cross-sectional studies (Mailing et al., 2019). For example, professional rugby players were found to have greater alpha diversity (Clarke et al., 2014), and amino acid, carbohydrate metabolic pathways, and greater fecal short chain fatty acids (SCFA) (Barton et al., 2018) than sedentary individuals. Moreover, even in the non-athletic populations, women who exercised for at least 3 hours per week had increased levels of butyrate producers such as *Faecalibacterium prausnitzii, Roseburia hominis*, and mucin degrader, *Akkermansia muciniphila*. However, the cross-sectional nature of these studies limits the ability of these studies to control diet and other confounding factors on the microbiome (Mailing et al., 2019).

The mechanisms by which exercise can impact the gut microbiome are not yet fully understood, but several potential mechanisms have been proposed (Mailing et al., 2019). The gut-associated lymphoid tissue (GALT) houses a majority of our immune cells, and exercise can modulate their gene expression, favoring anti-inflammatory and antioxidant profiles (Hoffman-Goetz et al., 2009; Hoffman-Goetz et al., 2010; Packer and Hoffman-Goetz, 2012), potentially mediating host-microbial interactions (Ismail et al., 2011; Mailing et al., 2019). Exercise can also influence the gut’s mucus layer, a critical barrier between microbes and the gut lining; as well as gut motility which can alter GI transit time, potentially impacting microbial habitats and their nutrient availability (Mailing et al., 2019). Furthermore, exercise can modify the circulation of bile acids (Meissner et al., 2011), known regulators of the microbial community structure (Kakiyama et al., 2013; Mailing et al., 2019). Finally, the metabolic demands of exercise release various compounds like lactate and myokines (Egan and Zierath, 2013) that might directly or indirectly interact with the gut environment (Mailing et al., 2019) potentially influencing the microbiome. Overall, the impact of exercise on the gut microbiome is likely mediated by a combination of these mechanisms. However, the magnitude and consistency of these effects are still unclear, and there is significant variation in study designs, including differences in exercise types, durations, intensities, and populations studied, as well as confounding effects of uncontrolled dietary and environmental factors.

To address these issues, this systematic review aims to synthesize the available data on the effects of exercise on gut microbiome in humans and animal models. The study of the impact of exercise on the animal microbiome is important because it can provide valuable mechanistic insights into the complex interactions between host physiology, behavior, and the gut microbiota. Additionally, animal models allow for greater experimental control of variables such as diet and genetics, which can be difficult to control in human studies. More specifically, the systematic review examines the impact of exercise type, duration, and intensity on changes in gut microbiome outcomes such as microbial phyla and genera composition, alpha- and beta-diversity, and microbial-produced metabolites such as SCFAs, etc., in trials with 2 or more arms, in both human and animal models. The results of this review will provide a better understanding of the relationship between exercise and the gut microbiome, potentially identifying targets for interventions to improve gut and metabolic health.

## 2 Methods

The study protocols for the human review is registered with PROSPERO, the International Prospective Register of Systematic Reviews (CRD42023394223).

### 2.1 Search strategy

A search strategy was designed based on guidelines in the Cochrane Handbook of systematic reviews (Cochrane Handbook for Systematic Reviews of Interventions, 2023). Specific terms and language were utilized to classify and analyze studies that included exercise and assessment of the gut microbiome. Search keywords are listed in Supplementary Table S1. Primary searches were conducted until 6 June 2022 to obtain studies that were published in the interim. Following this, the protocol was registered, and data extraction began. There are no restrictions on language, study design, or date of publication.

### 2.2 Study selection

The study search was performed across 4 databases, PubMed, Web of Science, Scopus, and Cochrane. Results were then collected in Zotero (version 5.0.87). To determine inclusion of studies in the review, three authors (JD, JC, SS) reviewed the articles in a methodical manner. Any disparities were decided through an inclusion vote. The PICO framework is described in Supplementary Table S2.

In the first pass, titles were independently screened to identify relevant articles based on specific criteria. Articles were excluded if the studies were 1) duplicates, 2) reviews, perspectives, or protocol papers, 3) published as conference abstracts, 4) not related to either exercise or the gut microbiome, or 5) were in children or pregnant populations. In the second pass, abstracts were screened for the same criteria in the first pass with additional criteria to exclude studies that were 1) either observational or cross-sectional, 2) not exercise interventions, and 3) did not have full texts. In the third pass, full texts were screened for the same criteria in the first two passes with additional criteria to exclude studies that 1) did not include comparator groups, 2) collected gut microbiome data at one time point only, 3) studies where exercise interventions were not clearly defined. Lastly, articles were categorized based on human studies and animal studies (mouse, rat, and horse).

### 2.3 Data extraction

Data collection tables were developed and finalized by 3 authors (JD, JC, SS). Extraction of data was performed by 2 authors (JC, SS) and was reviewed for accuracy by JD. Study quality was evaluated using the Oxford Centre for Evidence-Based Medicine Levels of Evidence (Centre for Evidence-Based Medicine, 2009). The risk of bias analysis was conducted using the quality control checklists of the Academy of Nutrition and Dietetics (Academy of Nutrition and Dietetics, 2022).

## 3 Results

**FIGURE 1:**
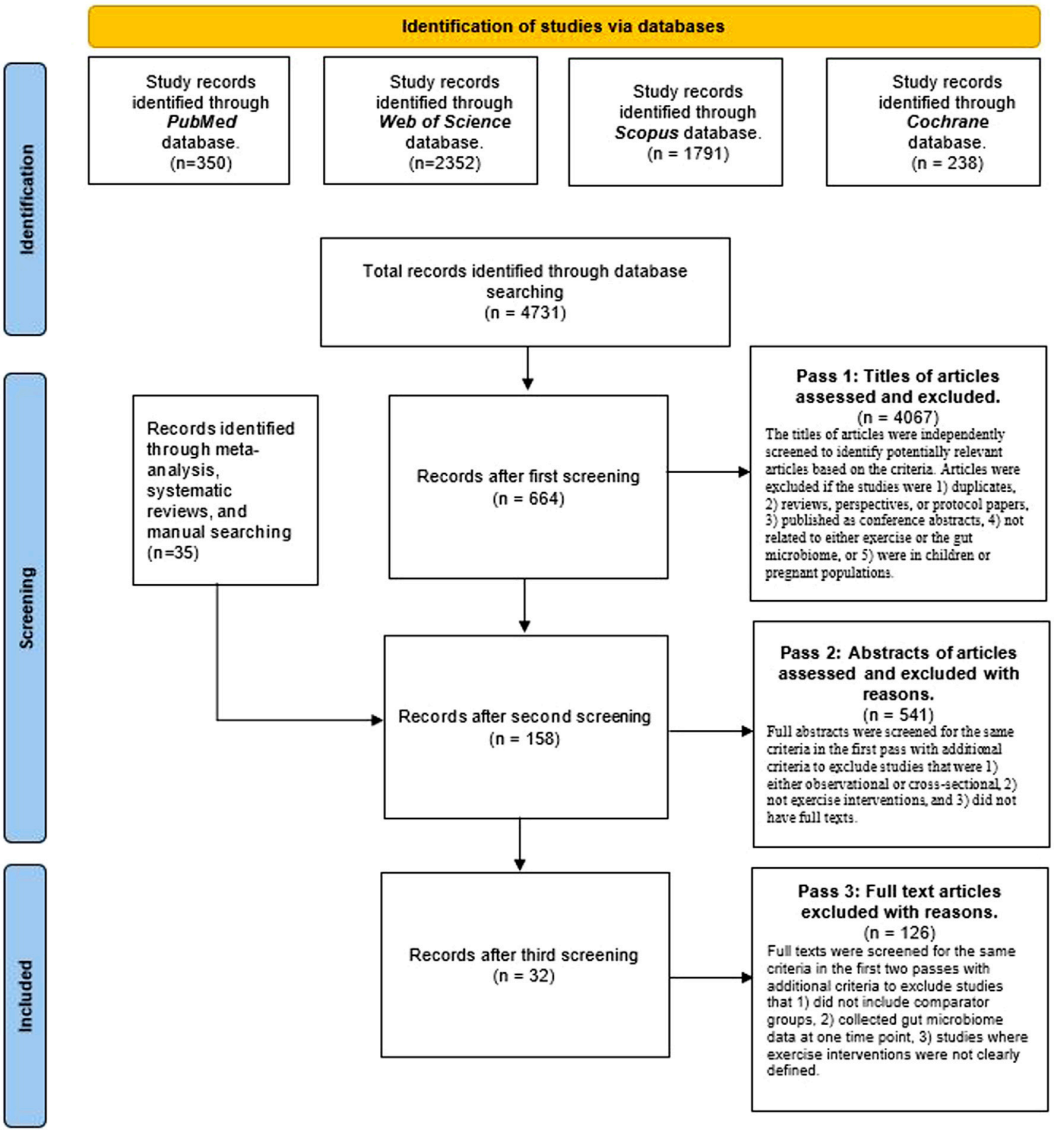
PRISMA flow diagram for inclusion of studies in the systematic review.

**FIGURE 2:**
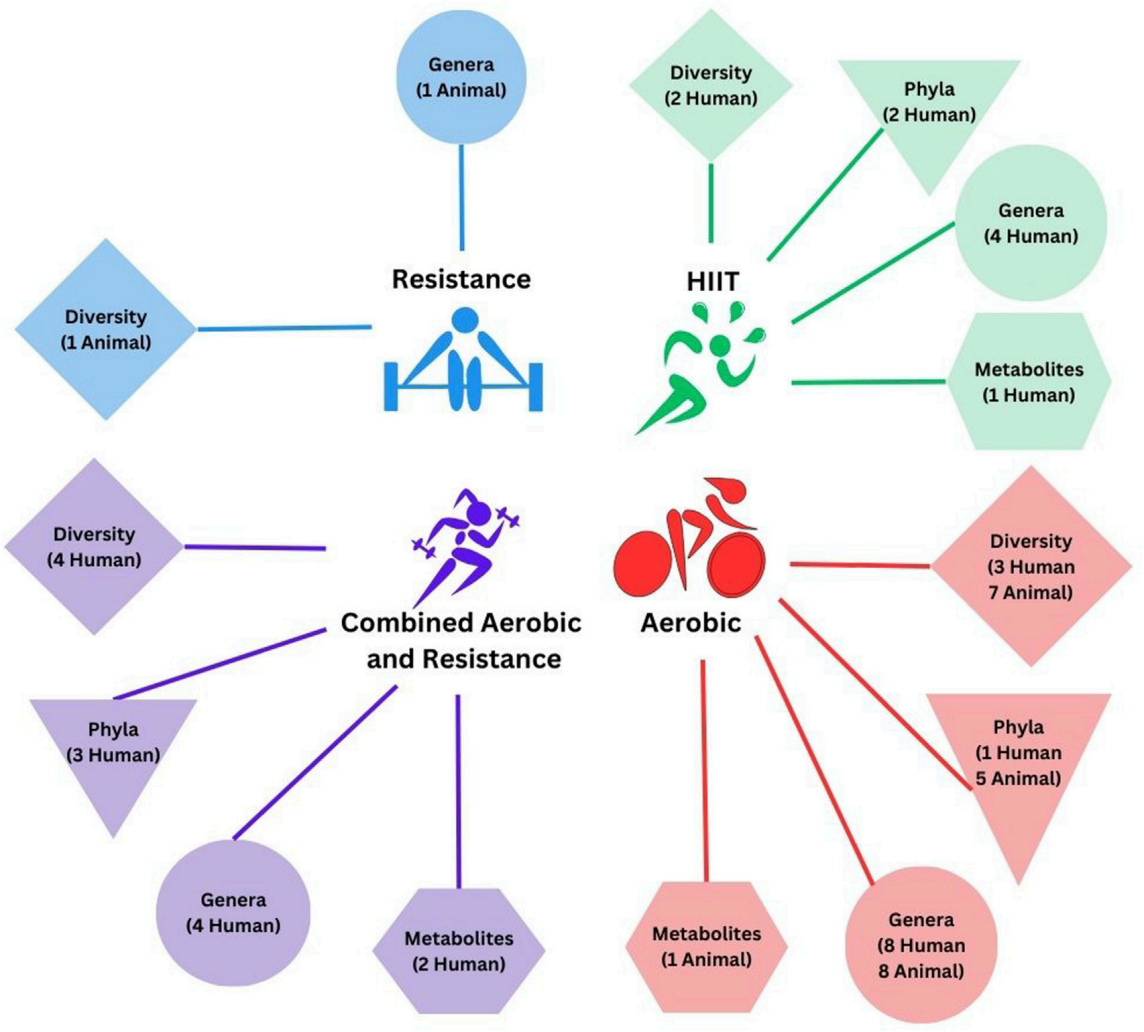
Microbiome outcomes stratified by exercise intervention type. The number indicates the number of studies that demonstrate a change in response to the exercise intervention in humans or animal models. Direction of change is not quantified. Study interventions incorporating HIIT or HIIT + another exercise modality are stratified separately.

In total, 4,731 records were screened for inclusion from database searches and 35 from meta-analyses and systematic reviews and manual search of reference lists. A total of 4,608 records were excluded based on the established criteria (Figure 1). Thirty-two studies, i.e., 19 human studies, and 13 animal studies with 2 or more groups that discussed microbiome outcomes, such as diversity, taxonomic composition, or microbial metabolites, over the intervention period, were included in the systematic review. The detailed inclusion and exclusion criteria are presented in Figure 1. The breakdown of outcomes by exercise intervention type is depicted in Figure 2.

### 3.1 Intervention type, study design, duration, and population profiles

Nineteen studies conducted in humans met our inclusion criteria. Of those, 11 included aerobic training interventions (Taniguchi et al., 2018; Morita et al., 2019; Resende et al., 2021; Moitinho-Silva et al., 2021; Mahdieh et al., 2021; Cheng et al., 2022; Sun et al., 2019; Calabrese et al., 2022; Motiani et al., 2020; Kern et al., 2020; Warbeck et al., 2021), 2 included resistance training interventions (Morita et al., 2019; Moitinho-Silva et al., 2021) and 9 included combined aerobic and resistance training interventions (Cronin et al., 2018; Cronin et al., 2019; Liu et al., 2020; Mokhtarzade et al., 2021; Zhong et al., 2020; Calabrese et al., 2022; Dupuit et al., 2022; Torquati et al., 2022; Wei et al., 2022). Six studies included a high intensity interval training intervention (Liu et al., 2020; Motiani et al., 2020; Warbeck et al., 2021; Dupuit et al., 2022; Sun et al., 2022; Torquati et al., 2022) Included studies varied in design, duration, and populations studied (Supplementary Table S3). Seventeen studies were trials with a parallel design (2–6 arms) (Cronin et al., 2018; Morita et al., 2019; Kern et al., 2020; Liu et al., 2020; Motiani et al., 2020; Moitinho-Silva et al., 2021; Mokhtarzade et al., 2021; Resende et al., 2021; Warbeck et al., 2021; Zhong et al., 2020; Calabrese et al., 2022; Cheng et al., 2022; Dupuit et al., 2022; Sun et al., 2022; Torquati et al., 2022; Wei et al., 2022; Mahdieh et al., 2021), of which the longest trial had a duration of 12 mon (Wei et al., 2022) and the shortest trials had a duration of 2 weeks (Motiani et al., 2020). One study followed a crossover design in which participants were exposed to a 5 weeks exercise intervention and a 5 weeks control (Taniguchi et al., 2018). Another study was an 8-week RCT with a 2- arm parallel design with a partial crossover (Cronin et al., 2019). The studies involve a diverse range of participant profiles, including individuals with prediabetes or type 2 diabetes, older males and females, individuals with normal blood pressure and non-diabetic status, those with inflammatory bowel disease (IBD), multiple sclerosis (MS), nonalcoholic fatty liver disease (NAFLD), and celiac disease, as well as individuals with overweight and obesity (Supplementary Table S3).

Thirteen studies conducted in various animal models met the inclusion criteria for this review (Supplementary Table S4). Of these 13, 5 were conducted in mice (Lamoureux et al., 2017; Houghton et al., 2018; Ribeiro et al., 2019; Yang et al., 2021; Chen et al., 2021), 4 were in rats (Mika et al., 2015; Giacco et al., 2020; Meng et al., 2020; Castro et al., 2021), and 4 in horses (De Almeida et al., 2016; Janabis et al., 2016; Janabi et al., 2017; Walshe et al., 2021). All but one (Castro et al., 2021) of these studies examined the effects of either aerobic exercise or interventions with aerobic exercise as an important component. Twelve studies were trials with a parallel design (2–8 arms) (Mika et al., 2015; De Almeida et al., 2016; Janabis et al., 2016; Lamoureux et al., 2017; Houghton et al., 2018; Ribeiro et al., 2019; Giacco et al., 2020; Meng et al., 2020; Yang et al., 2021; Castro et al., 2021; Chen et al., 2021; Walshe et al., 2021), of which the longest trial had a duration of 7 mon (Houghton et al., 2018) and the shortest trials had a duration of 3 days (Giacco et al., 2020). One study followed a crossover design in which horses were exposed to a graded exercise test and a standing control (Janabi et al., 2017). Most mice studies comprised of male and female C57BL/6 mice aged between 3–10 weeks. Rats used in the studies were male Wistar rats aged between 6–13 weeks (Giacco et al., 2020; Castro et al., 2021), Sprague Dawley rats with obesity aged 16 weeks (Meng et al., 2020), and adult male Fischer F344 rats (Mika et al., 2015). Standardbred horses, including mares and geldings, aged 3–17 years old were the most common horse breed used in the studies.

### 3.2 Effects of exercise interventions on microbial diversity

#### 3.2.1 Human studies

Sixteen intervention studies in humans examined microbial diversity outcomes. In a sedentary but healthy population of males and females, 6 weeks of aerobic training 3 times a week, but not resistance training decreased Chao1 index (Moitinho-Silva et al., 2021). In a similar population, 6 months of moderate and vigorous intensities of leisure time exercise and cycling 5x/week resulted in a greater shift in Bray-Curtis dissimilarity compared to the non-active control (Kern et al., 2020). Additionally, the cycling group showed a greater change in weighted unifrac diversity while the vigorous intensity group showed an increase in Shannon’s index after 3 months (Kern et al., 2020).

Two studies incorporated exercise with dietary interventions. In one study, combined aerobic and resistance training 3 times a week for 8 weeks along with 24 g protein consumption resulted in lower archeal Shannon’s index but higher bacterial Shannon’s index when compared to the protein only group, and in comparison to the exercise only group, lower viral Shannon’s index at week 8 (Cronin et al., 2018). All three groups showed separation in species Bray-Curtis dissimilarity after the 8 weeks intervention (Cronin et al., 2018). In the other study, a one-year lifestyle intervention resulted in an increase in overall richness and Shannon’s index in individuals with type 2 diabetes which was not different from the standard care control (Wei et al., 2022)*.* The only changes unique to the lifestyle intervention groups were observed differences in weighted unifrac measures at 3 months, however this difference between groups was not maintained throughout the whole intervention (Wei et al., 2022).

In a similar population with diabetes, moderate-intensity continuous training vs. high-intensity exercise interval training (combined aerobic and resistance) resulted in differential effects on Euclidean diversity between the two groups after 8 weeks of training (Torquati et al., 2022). In postmenopausal females with overweight and obesity, concurrent HIIT and resistance training for 12 weeks resulted in greater change of unweighted unifrac diversity compared to the control (Dupuit et al., 2022). Nine studies demonstrated no change in alpha or beta-diversity with exercise training (Taniguchi et al., 2018; Cronin et al., 2019; Liu et al., 2020; Motiani et al., 2020; Resende et al., 2021; Warbeck et al., 2021; Zhong et al., 2020; Calabrese et al., 2022; Sun et al., 2022).

#### 3.2.2 Animal studies

Thirteen intervention studies in animal models examined microbial diversity outcomes.

Three mice studies captured differences in diversity outcomes. Aerobic exercise 5x/week resulted in lower Chao1 index in male C57BL mice when compared to the sedentary control after 14 weeks (Yang et al., 2021). In another study that examined the effects of exercise in response to aging over the course of 7 months found that PolgA mut/mut mice undergoing aerobic exercise 4x/week had differential effects in Bray-Curtis dissimilarity compared to PolgA mut/mut sedentary (Houghton et al., 2018). Aerobic exercise of various durations (20 min, 40 min, and 60 min) resulted in greater observed OTU’s, Shannon’s index, lower Simpson’s index, and differential Bray-Curtis dissimilarity in female C57BL/6 mice at 4 weeks compared to control that had unrestricted access to run (Chen et al., 2021).

Two rat studies captured differences in diversity outcomes. In one study that incorporated aerobic exercise at different temperature conditions found that exercise only at room temperature resulted in increased Shannon’s index over 5 weeks, and exercise alone at room temperature vs. control resulted in differential alterations in bray-curtis and weighted unifrac at 5 weeks (Meng et al., 2020). A 12-week resistance training intervention resulted in greater Chao1 index and differential unweighted unifrac diversity in comparison to control after 12 weeks (Castro et al., 2021).

Three horse studies captured differences in diversity outcomes. In one study, a twelve-week aerobic exercise training intervention decreased Shannon’s index at the phyla level after 2 weeks of intervention which then increased from week 4 to week 6 (Janabis et al., 2016). In another study, aerobic exercise resulted in differential Jaccard diversity at 6 weeks in comparison to control (De Almeida et al., 2016). In a weight-loss intervention, aerobic exercise for 6 weeks increased Fisher alpha index, richness index, and Simpson index (Walshe et al., 2021).

Five studies demonstrated no change in alpha or beta-diversity with exercise training (Mika et al., 2015; Janabi et al., 2017; Lamoureux et al., 2017; Ribeiro et al., 2019; Giacco et al., 2020).

### 3.3 Effects of exercise interventions on microbial phyla and genera composition

#### 3.3.1 Human studies

Overall, fifteen studies in humans examined taxonomic composition at the phylum and genus levels. Four studies were conducted in healthy individuals. In healthy males, 10 weeks of cycling resulted in increased abundance of the genus *Streptococcus* and an unidentified genus in the *Clostridiales* order compared to the non-exercising controls (Resende et al., 2021). In elderly females, brisk walking increased the genus *Bacteroides*, while trunk muscle training demonstrated no effects (Morita et al., 2019). In another study conducted in elderly females, a combined aerobic and resistance training program for 8 weeks decreased the phylum *Firmicutes* and increased the genera *Phascolarctobacterium* and *Mitsuokella* (Zhong et al., 2020)*.* In elderly males, a change to 5-week cycling intervention increased abundance of the genus *Oscillospira* compared to the control (Taniguchi et al., 2018).

Four studies were conducted in healthy individuals with overweight and obesity. A 10 weeks aerobic exercise training intervention in sedentary females increased genus *Bifidobacterium* in comparison to the control (Mahdieh et al., 2021). In a dietary intervention that incorporated exercise training, the low carbohydrate (LC) diet and aerobic exercise (moderate (MICT) and high intensity (HIIT)) groups exhibited lower abundance of genera *Alistipes* at 4 weeks compared to LC alone, while the LC-MICT had higher abundance of the genus *Blautia* compared to LC alone (Sun et al., 2022). The LC-HIIT group also decreased *Bifidobacterium* abundance over 4 weeks (Sun et al., 2022). Two studies did not find any effects of exercise training on phyla or genera composition (Kern et al., 2020; Dupuit et al., 2022).

Seven studies were conducted in populations with pre-diabetes, diabetes, NAFLD, IBD, Multiple Sclerosis (MS), or celiac disease.

In a T2D population, moderate-intensity combined aerobic and resistance training led to higher collective abundance of phyla *Verrucomicrobia, Actinobacteria,* and *Desulfobacterota,* and genus *Bifidobacterium* compared to high-intensity combined aerobic and resistance training after 8 weeks (Torquati et al., 2022). In a prediabetic and diabetic population, both moderate intensity continuous training and sprint interval training for 2 weeks decreased the Firmicutes/Bacteroidetes ratio driven by an increase in Bacteroidetes, and decreased genera *Blautia* and *Closridium*, while only the moderate continuous training group increased genera *Veillonella* and *Faecalibacterium*, and only sprint interval training increased genus *Lachnospira* (Motiani et al., 2020). In a diabetic population, a 12-month lifestyle intervention consisting of dietary counseling and exercise decreased the Firmicutes/Bacteroidetes ratio and increased abundance of genera *Bacteroides* and *Roseburia* similar to the standard of care (Wei et al., 2022).

In individuals with NAFLD, the aerobic exercise only group exhibited greater genera *Bilophila, Roseburia, Erysipelotrichaceae_UCG_003, Hungatella, and Lachnospiraceae_UCG_004* whereas the aerobic exercise plus diet group exhibited greater genera *Alistipes*, *Bacteroides*, *Bilophilla, Butyricimonas, Roseburia,* and several genera in the *Lachnospiriceae and Ruminococcaceae* families (Cheng et al., 2022) after the intervention in comparison to the control. In another study, a low glycemic index Mediterranean diet in conjunction with an aerobic exercise intervention for 3 months led to higher abundance of genera *Ruminococcus, Lachnospiraceae_GCA900066575, Clostridia VadinBB60 group, Enterorabdus, Coprobacter, UCG002 (Oscillospiraceae), Intestinimonas, and Ruminococcaceae_g_UBA 1819*, and lower abundance of *Coprococcus* in individuals with NAFLD in comparison to the same diet and exercise alone groups (Calabrese et al., 2022).

In individuals with MS, a 6-month home-based combined aerobic and resistance training program increased genus *Prevotella* compared to a non-exercising control (Mokhtarzade et al., 2021). In individuals with celiac disease, a 12 weeks HIIT intervention enriched genera *Parabacteroides* and *Defluviitaleaceae_UCG_011* (Warbeck et al., 2021).

#### 3.3.2 Animal studies

Twelve intervention studies in animal models examined taxonomic composition at the phylum or genus levels.

All four studies in mouse models examined the effects of aerobic exercise on the gut microbiome. Aerobic exercise 5x/week resulted in higher abundance of phylum *Actinobacteria* and lower abundance of genera *Bacteroides, Parabacteroides,* and *Odoribacteria* in male C57BL mice compared to control group after 14 weeks (Yang et al., 2021). Moreover, the exercise group showed an increase in the genera *Bifidobacterium*, *Coprococcus*, and an unidentified genus in the *Clostridiales* order over 14 weeks (Yang et al., 2021). In a similar model of male C57BL mice, the effects of a high fat diet were compared to a standard chow diet with and without 8 weeks of aerobic exercise (Ribeiro et al., 2019). They only observed changes at the genus level with both exercise groups exhibiting higher *Vagococcus* and lower *Proteus* abundances after the training period when compared to their non-exercising controls. In another study, aerobic exercise led to higher phyla *Bacteroidetes* and *Verrucomicrobia* and lower *Firmicutes* over 4 weeks (Chen et al., 2021). At the genus level, exercise led to higher *Akkermansia, Clostridium, Parabacteroides, Christensenella, Dorea, Roseburia*, and *Paraprevotella* and lower *Anaerotruncus, Jeotgalicoccus, Flexispira, Alistipes, Ruminococcus*, and *Desulfovibrio* compared to the control at 4 weeks (Chen et al., 2021). In the PolgA mut/mut mice study (Houghton et al., 2018), aerobic exercise for 7 months increased the Firmicutes:Bacteriodetes ratio and the genus *Bacteroides.* Additionally, the exercise groups exhibited a higher abundance of the genera *Muucispirillum* and *Desulfovibrio* when compared to the non-exercised controls at 7 months (Houghton et al., 2018).

Four studies conducted in rats examined the effects of exercise training on phyla or genera outcomes. A 12 weeks resistance training intervention in male Wistar rats resulted in decreased *Pseudomonas*, *Serratia*, *Comamonas* but increased *Coprococcus_1* in comparison to the control (Castro et al., 2021). In the study that incorporated aerobic exercise at different temperature conditions, exercise at room temperature for 5 weeks resulted in increases in the genera *Parabacteroides*, *Ruminiclostridium*, *Allobaculum*, *Faecalibaculum*, *Faecalitalea*, *Holdemania*, and *Gelria* when compared to the non-exercising control (Meng et al., 2020). Exercise with different modalities of cold exposure also resulted in other changes at the phylum and genera level which were not consistent for exercise as whole (Meng et al., 2020). Moreover, in a voluntary wheel running study, exercise for 6 weeks resulted in increased *Rikenellaceae* family genera *AF12* and an unclassified genus, and *Turicibacter* (Mika et al., 2015). One rat study did not find any effect of exercise training on phylum or genus taxonomic composition (Giacco et al., 2020).

Four studies conducted in horse models tested the effects of aerobic exercise and/or other lifestyle interventions on phyla or genera outcomes. In a weight-loss intervention, aerobic exercise led to higher abundance of the genus *Coprococcus* compared to the non-exercising control group at 6 weeks (Walshe et al., 2021). In another study, a 12 weeks aerobic exercise intervention led to an increase of the phylum *Bacteroidetes* in the first 2 weeks and then a decrease from week 4 to week 6 (Janabis et al., 2016). A progressive increase in the phylum *Proteobacteria* was also observed from week 4 to week 6 to week 8, and in the phylum *Spirochaetes* from week 2 to week 4, however there was a reduction in *Spirochaetes* from week 8 to week 10 in response to exercise. At the genus level, *Clostridium* increased from week 0 to week 2 and decreased from week 2 to week 4, whereas *Dysgonomonas* increased from week 2 to week 4 and decreased from week 4 to week 6 in response to exercise. Two horse studies did not find any effect of exercise training on phyla or genus taxonomic composition (De Almeida et al., 2016; Janabi et al., 2017).

### 3.4 Effects of exercise interventions on microbial metabolites

#### 3.4.1 Human studies

Only three human studies assessed effects of exercise training on gut microbiome derived metabolites. High-intensity combined aerobic and resistance training for 12 weeks in males with overweight and obesity resulted in a decrease in fecal and serum BCAA and aromatic AA, and an increase in fecal propionate and GABA, and serum SCFA in exercise responders (Liu et al., 2020). Additionally, Cronin et al. (2018) found that the a combined aerobic resistance training program of moderate intensity for 8 weeks resulted in a greater decrease in urinary PAG and TMAO compared to the protein only group. A similar study did not find any effects of exercise training on fecal SCFAs in individuals with type 2 diabetes (Torquati et al., 2022).

#### 3.4.2 Animal studies

Only two animal studies assessed gut microbial metabolites. Four weeks of aerobic training of different durations (20,40, or 60 min) in female C57BL/6 WT mice resulted in lower serum D-Lac, LPS, and DAO in comparison to unrestricted running control (Chen et al., 2021). However, no differences in fecal metabolomics profiles were found with aerobic training in overweight or obese mixed breed horses undergoing weight-loss (Walshe et al., 2021).

### 3.5 Risk of bias and quality of assessment of studies included in systematic review

Characteristics of the included human studies are summarized in Table 1. Seven articles were rated positive, indicating that they had adequately addressed issues of bias, generalizability, and data collection. The remaining 12 were found to be neutral, meaning they were neither exceptionally weak nor exceptionally strong. Characteristics of the included animal studies are summarized in Table 2. One article was rated as positive, 11 as neutral, and 1 as negative, indicating that it has inadequately addressed validity criteria. Overall, the human and animal studies lacked consistency in reporting of statistical analyses and interpretation of data. The issues with statistical analyses pertain to not reporting or including overall exercise group by time interaction effects, or adjusting post-intervention values for pre-intervention values in the statistical model, not accounting for repeated measures, restricting analysis to only selected exercise arms without specifying *a priori* hypotheses, insufficient description of the statistical model for interpretation of results, or interpretation based on *post hoc* tests even when interaction effects were not significant.

**TABLE 1:** Risk-of-bias analysis of studies included in the systematic review examining the effects of exercise interventions on the gut microbiome in humans.

Study	Selection of participants free from bias	Study with >80% follow up^a^	Standard/valid/reliable data collection procedures	QCC^b^ ^,^ ^c^ rating
Calabrese et al. (2022)	Yes	No	Yes	∅
Cheng et al. (2022)	Yes	No	Yes	∅
Cronin et al. (2018)	Yes	Yes	Yes	+
Cronin et al. (2019)	Yes	No	Yes	∅
Dupuit et al. (2022)	Yes	No	Yes	∅
Kern et al. (2020)	Yes	No	Yes	∅
Liu et al. (2020)	Yes	Yes	Yes	∅
Mahdieh et al. (2021)	Yes	Yes	Yes	+
Moitinho-Silva et al. (2021)	Yes	Yes	Yes	∅
Mokhtarzade et al. (2021)	Yes	Yes	Yes	+
Morita et al. (2019)	No	Yes	Yes	∅
Motiani et al. (2020)	Yes	No	Yes	+
Resende et al. (2021)	Yes	Yes	Yes	+
Sun et al. (2022)	Yes	No	Yes	∅
Taniguchi et al. (2018)	Yes	Yes	Yes	+
Torquati et al. (2022)	Yes^d^	No	Yes	∅
Warbeck et al. (2021)	Yes	No	Yes	∅
Wei et al. (2022)	Yes	No	Yes	∅
Zhong et al. (2020)	Yes	Yes	Yes	+

^a^ Follow-up here takes into account participant attrition or participants included in analysis.

^b^ QCC, quality criteria checklist.

^c^ QCC, rating: +, report has clearly addressed issues of inclusion/exclusion, bias, generalizability, and data collection and analysis; ∅, report is neither exceptionally strong nor exceptionally weak.

^d^ Original study.

**TABLE 2:** Risk-of-bias analysis of studies included in the systematic review examining the effects of exercise interventions on the gut microbiome in animal models.

Study	Selection of participants free from bias	Study with >80% follow up^a^	Standard/valid/reliable data collection procedures	QCC rating^b^ ^,^ ^c^
Castro et al. (2021)	Yes	Yes	Yes	∅
Chen et al. (2021)	Yes	Unclear	Yes	-
De Almeida et al. (2016)	Yes	Yes	Yes	+
Giacco et al. (2020)	Yes	No	Yes	∅
Houghton et al. (2018)	Yes	Unclear	Yes	∅
Janabi et al. (2017)	Yes	Yes	Yes	∅
Janabis et al. (2016)	Yes	Yes	Yes	∅
Lamoureux et al. (2017)	Yes	Unclear	Yes	∅
Meng et al. (2020)	Yes	Yes	Yes	∅
Mika et al. (2015)	Yes	No	Yes	∅
Ribeiro et al. (2019)	Yes	No	Yes	∅
Walshe et al. (2021)	No^d^	Yes	Yes	∅
Yang et al. (2021)	Yes	Unclear	Yes	∅

^a^ Follow-up here takes into account participant attrition or participants included in analysis.

^b^ QCC, quality criteria checklist.

^c^ QCC, rating: +, report has clearly addressed issues of inclusion/exclusion, bias, generalizability, and data collection and analysis; -, report has not addressed these issues adequately; and ∅, report is neither exceptionally strong nor exceptionally weak.

^d^ Client-owned, not experimental animals.

## 4 Discussion

### 4.1 Summary of evidence

More than 50% of the studies did not demonstrate any exercise effects on microbial diversity in humans. Of those that did, exercise was found to generally favor an increase in Shannon’s index, an index of microbial richness and evenness, regardless of disease status. However, when combined with dietary changes, the impact on alpha-diversity can vary. For instance, one study demonstrated opposite effects for archaeal versus bacterial Shannon’s index in response to a combined aerobic and dietary protein intervention (Cronin et al., 2018). The weighted unifrac measure, a phylogenetic measure that accounts for the relatedness of microbial taxa, was the most commonly reported beta-diversity metric. Changes in other beta-diversity measures, like Bray-Curtis dissimilarity, both weighted and unweighted Unifrac, and Euclidean diversity, were also documented regardless of disease status and exercise type. The direction of these changes, however, was not specified. A greater proportion of animal studies reported changes in diversity compared to human studies. However, no clear pattern emerged due to the mixed effects of exercise (predominantly aerobic) on alpha or beta-diversity metrics.

According to ecological theory, diversity in the gut microbiome, both in terms of species richness and functional response, is integral to its resilience (Lozupone et al., 2012). A diverse microbiome is better equipped to resist invaders and efficiently use resources. Notably, even when certain species are compromised, others, with similar functions, can step in, ensuring stability. This phenomenon is further demonstrated by the functional redundancy seen in various bacteria in the gut. Thus, a richer and functionally diverse microbiome may offer greater stability and adaptability in the face of disturbances (Lozupone et al., 2012). It is plausible that, much like research on diet and the microbiome (Johnson et al., 2020a), the effects of an exercise intervention may be predicated upon the baseline microbiome composition of individuals, which in turn can be influenced by environmental factors, early life exposure, disease status, etc. (Dominguez-Bello et al., 2010; Vangay et al., 2015; Rothschild et al., 2018). Whether individuals with a less diverse or less stable microbiome at baseline may be more susceptible to changes in microbial composition with exercise compared to individuals with a more diverse and stable microbiome is a hypothesis that should be tested further. And even if overall diversity does not change significantly with exercise, individual taxa can still be impacted in terms of their relative abundance or metabolic activity.

Our review indicates that exercise interventions can also affect taxonomic composition at the phylum and genus levels. In humans, at the phyla level, exercise was typically associated with a decrease in the Firmicutes/Bacteroidetes ratio. At the genera level, while *Bacteroides*, *Roseburia*, *Blautia*, *Alistipes* were most reported to change in response to exercise in humans, only *Bacteroides* and *Roseburia*, demonstrated consistent increases with exercise. *Bacteroides* is a genus involved in many metabolic pathways including sphingolipid production (Johnson et al., 2020b), SCFA production, bile acid metabolism (Ghosh et al., 2021), and mucus utilization (Wang et al., 2023). *Roseburia* is majorly involved in the production of SCFAs, particularly butyrate, and in conjugated fatty acid metabolism (Ghosh et al., 2021). Both SCFA and conjugated fatty acids, play a role in regulation of intestinal permeability, while SCFA are also associated with energy homeostasis, and increased glucose tolerance and insulin sensitivity. Amongst the other genera that showed no consistent effects with exercise, *Blautia* has been associated with potential probiotic capabilities but is also enriched in IBD (Liu et al. 2021), *Alistipes* has been found to be pathogenic in colorectal cancer but has protective effects in CVD (Parker et al., 2020), while *Parabacteroides* is implicated in colitis (Dziarski et al., 2016) but also demonstrates a protective effect in obesity (Wang et al., 2019).

In animal models collectively, *Parabacteroides, Coprococcus*, *Bacteroides*, and *Clostridium* were most reported to change in response to exercise. However, only *Coprococcus*, another SCFA producer, demonstrated consistent increases with exercise. In general, in both human and animal models, the diversity in intervention duration, exercise modality and intensity, disease status, dietary factors, baseline microbiota composition, etc. appear to have a huge effect on individual microbial taxa, and due to the limited number of studies, stratification by these factors was not possible. A common pattern though was an exercise-induced increase in SCFA producers such as *Coprococcus*, *Dorea*, *Roseburia*, *Paraprevotella*, *Ruminiclostridium*, and *Allobaculum*. Our review of the literature also identified SCFA as the most measured metabolite. However, due to the limited human and animal studies examining exercise effects on microbial-produced metabolites including SCFAs, no clear patterns emerged.

### 4.2 Strengths and limitations of review

While a few systematic reviews on this topic have been published (Mailing et al., 2019; Mitchell et al., 2019; Tzemah Shahar et al., 2020; Clemente et al., 2021), the uniqueness of this systematic review lies in the inclusion of only studies that have a control or other arm for rigorous comparisons to the intervention arms, and inclusion of both human and animal studies for a comprehensive overview of the exercise-microbiome literature. However, like any field in the initial stages, the heterogeneity of exercise intervention designs, taxa outcomes profiled, and populations recruited, limited the ability to make direct comparisons among studies. Moreover, several studies included in this review did not account for confounding factors such as dietary habits, medication use, or other environmental factors, that could influence the relationship between exercise and the microbiome.

### 4.3 Implication of findings, research gaps and future directions

This research underscores the importance of the gut microbiome in personalized health and precision medicine by providing insights into the potential benefits of exercise on gut microbial diversity and composition. However, the lack of standardized exercise protocols across studies makes it challenging to compare results and draw conclusive findings. Establishing standardized protocols would enable better comparisons and generalizability of results. Additionally, there is a need for adequately powered studies and rigorous statistical models to accurately interpret group by time effects in longitudinal studies. In the context of precision medicine, considering factors such as disease status, exercise modality/intensity, and baseline microbiota composition can optimize the selection and customization of exercise interventions, leading to more effective therapeutic strategies for improving gut health.

An important research gap in the exercise-microbiome field is the limited utilization of interspersed designs that incorporate baseline microbiome composition. Currently, the typical practice is to employ randomization without considering the initial microbial profiles of participants. However, accounting for baseline microbiome composition or stratifying participants based on baseline microbiome composition ranges would capture a broader range of responses to exercise interventions and allow for assessing how these responses depend on the presence of selected microbes (Johnson et al., 2020a). Understanding an individual’s baseline microbiome composition and its response to exercise is crucial for personalized health. Individuals with a less diverse or stable microbiome at baseline may be more susceptible to changes in microbial composition with exercise, emphasizing the importance of tailored interventions to optimize gut health benefits. Implementing such designs would also improve statistical power.

Furthermore, the identification of specific genera, such as *Bacteroides*, *Roseburia*, and *Coprococcus*, as consistent responders to exercise highlights their potential as targets for interventions and biomarkers of exercise-induced changes in the gut microbiome. Future research should focus on elucidating the underlying mechanisms of these changes and their impact on health outcomes. While changes in microbial composition have been widely studied, investigating the specific microbial metabolites influenced by exercise is crucial to unravel the functional implications of exercise-induced microbiome changes. To gain a better understanding of the variability in responses and potential mechanistic insights, it is important to consider various factors such as employing “omics” techniques to examine biological factors and taking into account the socioecological context. Other key considerations in microbiome research from other reviews (Knight et al., 2018; Bharti and Grimm, 2021; Hughes and Holscher, 2021; Shanahan et al., 2021; Mattes et al., 2022), although mostly for diet studies, are also applicable to exercise studies.

## 5 Conclusion

In conclusion, this systematic review reveals that exercise has the potential to influence the gut microbiome, with indications of increased microbial diversity and changes in taxonomic composition. Further research is needed to standardize exercise protocols, incorporate larger sample sizes, and employ rigorous statistical models. Additionally, investigating the functional implications of exercise-induced changes in microbial metabolites and understanding the underlying mechanisms will enhance our understanding of the exercise-microbiome relationship. Considering the growing prevalence of chronic diseases, such as obesity, diabetes, and cardiovascular disorders, studying the exercise-microbiome relationship may provide valuable insights for preventive and therapeutic interventions. Targeted exercise programs that promote a favorable microbial profile could potentially serve as a non-invasive and cost-effective approach to support overall health and mitigate the risk of chronic diseases. These advancements can guide the development of personalized exercise interventions for optimizing health.

## Data Availability

The original contributions presented in the study are included in the article/Supplementary Material, further inquiries can be directed to the corresponding author.
