# Supplementary material for: A systematic review on the effects of exercise on gut microbial diversity, taxonomic composition, and microbial metabolites: identifying research gaps and future directions
Source: Front Physiol. 2023 Dec 19;14:1292673. doi: 10.3389/fphys.2023.1292673 (PMC10770260; doi:10.3389/fphys.2023.1292673)
Supplement: Supplementary file 2 [file Table2.docx]

**Supplemental Table 2**. PICO framework guiding the systematic review

|  | Human | Animal |
| --- | --- | --- |
| Population | Inclusion: Human adult populations (over 18 years old); non-pregnant populations | Inclusion: All animals  Exclusion: pregnant/very early life stage models; human models; ex vivo studies |
| Intervention | Exercise intervention trials that examine the gut microbiome. | Exercise intervention trials that examine the gut microbiome. |
| Comparison | Controls, dietary intervention groups, or different modalities of exercise groups | Controls, dietary intervention groups, or different modalities of exercise groups |
| Outcomes | Gut microbiome (diversity, phyla, and genera). SCFAs and other gut microbiome-derived metabolites | Gut microbiome (diversity, phyla, and genera). SCFAs and other gut microbiome-derived metabolites |
