# Supplementary material for: A systematic review on the effects of exercise on gut microbial diversity, taxonomic composition, and microbial metabolites: identifying research gaps and future directions
Source: Front Physiol. 2023 Dec 19;14:1292673. doi: 10.3389/fphys.2023.1292673 (PMC10770260; doi:10.3389/fphys.2023.1292673)
Supplement: Supplementary file 3 [file Table3.docx]

**Supplementary Table 3.** Characteristics of the studies included in the systematic review examining the effects of exercise intervention on gut microbiome outcomes in humans.

| Author | Design | No. of participants | Intervention | Duration | Participant profile | Phylum | Genus | Diversity | Gut metabolites |
| --- | --- | --- | --- | --- | --- | --- | --- | --- | --- |
| Calabrese et al. 2022 | Randomized (6 groups) | 144 (original dataset) | Physical activity aerobic program (ATFIS_1, n=25) 3x/wk  50-60 min moderate-intensity treadmill walking, cycling, cross-training, and rowing @ 60–75% VO2 max  Physical activity combined aerobic and resistance program (ATFIS_2, n=23) 3x/wk  45 min moderate-intensity treadmill walking or cycling @ 60–75% VO2 max + resistance training (12 exercises, 3 sets)   Low Glycemic Index Mediterranean Diet (LGIMD, n=23)  LGMID + ATFIS_1 (n=27)  LGMID + ATFIS_2 (n=24)  Control diet (n=22): based on CREA-AN dietary guidelines | 12 wk | Males and females with moderate to severe NAFLD, age: 30-60 yr, BMI ≥ 25 kg /m2 | N/A | LGMID + ATFIS_1 (vs. LGMID and ATFIS_1): ↑Ruminococcus ↑Lachnospiraceae_GCA900066575 ↑Clostridia VadinBB60 group ↑Enterorabdus ↑Coprobacter ↑UCG002 (Oscillospiraceae) ↑Intestinimonas ↑Ruminococcaceae_g_UBA1819  ↓ Coprococcus | No change in alpha-diversity | N/A |
| Cheng et al. 2022 | Randomized (4 groups) | 115 | Aerobic exercise (AEX, n=29): Nordic brisk walking + stretching and other group exercises 2-3x/wk @60-75% VO2max for 30-60 min/session   Diet (n=28): fiber-enriched and low-carbohydrate   Aerobic exercise + Diet (AED, n=29)  No intervention (NI, n=29): current physical activity and eating habits | Average: 37 wk | Males and females with NAFLD and prediabetes, age 50-65 yr, Mean BMI: 26.9 kg/m2 | N/A | AEX vs. NI: ↑ Bilophila  ↑ Erysipelotrichaceae_UCG_003 ↑ Hungatella ↑ Lachnospiraceae_UCG_004 ↑ Roseburia  AED vs. NI: ↑ Alistipes ↑ Bacteroides ↑ Bilophila ↑ Butyricimonas ↑ Lachnospiraceae ND3007, NK4A136, UCG_004, and UCG_010 groups ↑ Roseburia  ↑ Ruminococcaceae NK4A214, UCG_002, and UCG_003 groups ↑ Ruminococcus_1 | NI vs. other groups: ↓ Shannon Index Changes in Weighted Unifrac | N/A |
| Cronin et al. 2018 | Randomized (2 groups). Total 3 groups. | 90 | Exercise only (E, n=30): Combined aerobic resistance training program of moderate intensity 3x/wk  Aerobic exercise: treadmill, crosstrainer, bike, or stepper (modified Borg RPE, 5 to 7/10) 18-32 min/session  Resistance exercise: 7 resistance machine based exercises @ 70% 1RM starting intensity  Protein only (P, n=30): usual levels of physical activity and whey protein (24 g)  Exercise+protein (EP, n=30): Same exercise program as exercise only group and whey protein (24 g) | 8 wk | Sedentary males and females, age: 18-40 yr, BMI: 22-35 kg/m2 | N/A | N/A | EP vs. P:  ↓ Archeal Shannon's index at 8 wk ↑ Bacterial Shannon's index at 8 wk  EP vs. E:  ↓ Viral Shannon's index at 8 wk  Separation in all species Bray-Curtis dissimilarity by E, EP, and P at 8 wk | E vs. P: Greater ↓ urine PAG and TMAO over 8 wk |
| Cronin et al. 2019 | Randomized (2 groups) + partial crossover | 17 | Exercise (n=8 (initial) + 7 crossed over from control): Combined aerobic resistance training program of moderate intensity 3x/wk  Aerobic exercise: treadmill, crosstrainer, bike, or stepper (modified Borg RPE, 5 to 7/10) 18-32 min/session  Resistance exercise: 7 resistance machine based exercises @ 70% 1RM starting intensity  Control (n=9): usual levels of physical activity, n=7 crossed over to exercise group after completion of control period | 8 wk | Sedentary males and females, age: 18-40 yr, BMI: 22-35 kg/m2, diagnosed with IBD but currently in disease remission | N/A | N/A | No change | N/A |
| Dupuit et al. 2022 | 2 groups | 29 | Exercise (n=14): Concurrent HIIT and resistance training 3x/week, 45 min  HIIT: repeated cycles of sprinting on bike for 8s followed by slow pedaling for 12s  Resistance: whole body, 10 exercises, 1-set circuit, with 8–12RM load  Control (n=15): No training | 12 wk | Post-menopausal sedentary women with overweight and obesity, BMI: 25-40 kg/m2, mean age: 59.9 yr | No change | N/A | Exercise vs. control: Greater change in unweighted Unifrac | N/A |
| Kern et al. 2020 | Randomized (4 groups) | 133 (original dataset) | Control (n=14*): habitual living  Bike (n=19*): active commuting by cycling 5x/wk  Mod (n=31*): moderate leisure time exercise @ 50% VO2 Peak 5x/wk  Vig (n=24*): vigorous leisure time exercise @ 70% VO2 Peak 5x/wk  *no. of completers | 26 wk | Sedentary males and females with overweight and obesity, normotensive, non-diabetic, age: 20–45 yr, BMI: 25–35 kg/m2 | N/A | No change | Vig vs. control:  ↑ Shannon's index over 12 wk only.  All exercise groups vs. control: greater change in Bray-Curtis dissimilarity over 12 and 26 wk.  Bike vs. control: greater change in weighted Unifrac over 12 wk only | N/A |
| Liu et al. 2020 | Randomized (2 groups) | 42 | Exercise (n=23): High-intensity combined aerobic and resistance training 3x/week for 70 min  10 min intervals each of high-intensity treadmill, high-intensity resistance and calisthenics exercises, and high-intensity stationary bike with 3-4 min recovery between interval sessions   Control (n=19): sedentary | 12 wk | Males with overweight and obesity (BMI>23 kg/m2), age: 20-60 yr, prediabetes or impaired fasting glucose | N/A | N/A | No change | Exercise responders over 12 wk: ↓ fecal BCAA, aromatic AA ↑fecal propionate and GABA  ↑ serum SCFA ↓serum BCAA and aromatic AA |
| Mahdieh et al. 2021 | Randomized (2 groups) | 18 | Exercise (n=9): aerobic exercise 3x/week for 30-45 min. Starting intensity @ 55-60% HRR with progression to 70-75%  Waitlist control (n=9) | 10 wk | Inactive females with overweight and obesity, BMI: 25-32 kg/m2, age:19-30 yr | N/A | Exercise and control over 4 wk: ↑ Lactobacillus   Exercise vs. control over 4 wk: ↑ Bifidobacterium | N/A | N/A |
| Moitinho-Silva et al. 2021 | Randomized (3 groups) | 42 | Endurance (n=13): 30 min run 3x/week  Strength exercise (n=12): whole body hypertrophy strength training 3x/week, 30 min/session  Control (n=11): Maintain usual physical activity | 6 wk | Sedentary but healthy males and females, age: 20-45 yr, BMI: 20-35 kg/m2 | N/A | N/A | Endurance: ↓ Chao1 | N/A |
| Mokhtarzade et al. 2021 | Randomized (2 groups) | 42 | Home based exercise (HBE, n=21): Combined aerobic exercise (3x/wk) and resistance exercise (2x/wk)  Aerobic exercise: 50-75% of HRR  Resistance exercise: elastic bands and body weight  Waitlist control (n=21): routine physical activity | 26 wk | Males and females with MS, age: 18-50 yr, expanded disability scale status<5 | N/A | HBE vs. control over 26 wk: ↑ Prevotella | N/A | N/A |
| Morita et al. 2019 | Non-randomized (two groups) | 32 | Aerobic exercise (AE, n=18): brisk walking 60 min daily @ intensity ≥ 3 METs  Trunk muscle training (TM, n=14): targeted resistance training of trunk muscles 1x/wk for 55-65 min/session | 12 wk | Healthy females, age > 65 yr, median BMI: 21.4 kg/m^2^ | N/A | AE: ↑ Bacteroides | N/A | N/A |
| Motiani et al. 2020 | Randomized (2 groups) | 26 | Sprint Interval Training (SIT, n=13): 4-6 30 s cycling bouts/session 3 x/wk  Moderate intensity continuous training (MICT, n=13):  40-60 min of cycling @ 60% VO2 max 3x/wk | 2 wk | Males and females with prediabetes or type 2 diabetes, age: 40-55 yr, BMI: 30.5 ± 3 kg/m^2^ | SIT and MICT over 2 wk: ↓ F/B ratio ↑ Bacteroidetes | SIT and MICT over 2 wk:  ↓ Blautia ↓ Clostridium  SIT over 2 wk: ↑ Lachnospira   MICT over 2 wk: ↑ Veillonella  ↑ Faecalibacterium | No change | N/A |
| Resende et al. 2021 | Randomized (2 groups) | 28 | Exercise (n=14): cycle ergometer 3x/wk for 50 min/session @ 60-65% VO_2_ peak  Control (n=14): normal lifestyle with no change to physical activity | 10 wk | Healthy men, age: 20-45 yr, BMI: 24.5 ± 3.7 kg/m^2^ | No change | Exercise vs. control over 10 wk: ↑ Streptococcus  ↓ Unclassified clostridiales order genus | No change | N/A |
| Sun et al. 2022 | Randomized (3 groups) | 50 | Low carb diet (LC, n=16):  dietary guidance for low carbohydrate diet  LC+HIIT (n=17): LC + cycling on cycle ergometer, sprinting for 6s against resistance + 9s rest x 10  LC+MICT (n=17): LC + 30 min cycling on cycle ergometer @ 50-60% of VO2 peak | 4 wk | Females with overweight and obesity (BMI >23 kg/m2), age: 21.6 ± 3.4 y | No change | LC: ↑ Phascolarctobacterium over 4 wk  LC-HIIT: ↓ Bifidobacterium over 4 wk  LC-MICT vs. LC at 4 wk:  ↑Blautia ↓Alistipes  LC-HIIT vs. LC at 4 wk: ↓Alistipes | No changes | N/A |
| Taniguchi et al. 2018 | Randomized crossover | 33 | Endurance: cycle ergometer 3x/wk. Intensity gradually inc. from 60% - 75% VO_2_ peak for 30-45 min/session  Control | 5 wk each phase | Healthy males, age >60 yr, mean BMI: 22.9 ± 2.5 kg/m^2^ | No change | Endurance vs. control over 5 wk: ↑ Oscillospira | No change | N/A |
| Torquati et al. 2022 | Randomized (2 groups*) *original study had 3 groups | 32 (original dataset) | Combined aerobic and resistance moderate intensity continuous training (C-MICT, n=7*): 4x/week for 52.5 minutes  Combined aerobic and resistance high intensity interval training ( C-HIIT, n=5*): 3x/week for 26 minutes  *no. of completers | 8 wk | Male and females with T2D, low activity levels, age: 64.3 ± 6.4 yr, Mean BMI: 34.6 kg/m2 | C-MICT vs. C-HIIT: ↑ Collective abundance of Verrucomicrobia, Actinobacteria, and Desulfobacterota after 8 wk | C-MICT vs. C-HIIT: ↑ Bifidobacterium after 8 wk | C-MICT vs. C-HIIT: Differences in Euclidean diversity after 8 wk | No changes in fecal SCFA |
| Warbeck et al. 2021 | Randomized (2 groups) | 41 | HIIT+ (n=20): HIIT 2x/week, 60 min sessions + group-mediated cognitive-behavioral intervention.  HIIT intervals: 30 s of vigorous effort (90% HRmax) followed by 2 min (50% HRmax) of recovery using stationary bikes, ellipticals, treadmills, or BW exercises  Wait list control (WLC, n=21) | 12 wk | Inactive males and females with celiac disease, age>18 yr, mean BMI: 27.9 kg/m2 | No change | HIIT+ vs. WLC enrichment analysis at 12 wk:  HIIT+ enriched in Parabacteroides and  Defluviitaleaceae_UCG_011  WLC enriched in Roseburia, Klebsiella and Adlercreutzia | No change | N/A |
| Wei et al. 2022 | Randomized (2 groups) | 98 | Standard care (SC, n=34): medical counseling, T2D education, lifestyle advice.  Lifestyle (n=64): SC + 5-6 exercise sessions/wk + dietary plan + diet counseling  Exercise: 5-6 aerobic sessions/wk of which 2-3 were combined with resistance training sessions, 30-60 min/session  Both groups encouraged to be physically active in leisure time (≥ 10,000 steps/day) | 52 wk | Males and females with T2D, age≥18 yr, BMI: 25-40 kg/m2 | Lifestyle and SC over 13 and 52 wk:  ↓F/B | Lifestyle over 13 wk and 52 wk: ↑ Bacteroides  ↑ Roseburia  SC:  ↑ Bacteroides over 13 wk  ↑ Roseburia over 52 wk | Lifestyle and SC over 52 wk:  ↑ Richness ↑ Shannon's index  Lifestyle over 13 wk only:  ↑ Shannon's index  Lifestyle vs. SC at 13 wk: Differences in weighted UniFrac | N/A |
| Zhong et al. 2020 | Randomized (2 groups) | 14 | Exercise (n=7): combined aerobic and whole body resistance exercise (elastic bands) 4x/wk for 60 min/session  Control (n=7): maintain daily life and watched health related videos 2x/month | 8 wk | Sedentary females, age: 60-75 yr, mean BMI: 22.96 kg/m^2^  HbA1C < 6.5%, fasting BG < 7.0mmol/L | Exercise: ↓Firmicutes   Control: ↑ Bacteroidetes ↓ Actinobacteria | Exercise: ↑ Phascolarctobacterium  ↑ Mitsuokella   Control: ↑ Bacteroides  ↑ Parabacteroides  ↓ Eubacterium | No change | N/A |

Results from human studies examining the effects of exercise on the gut microbiome

**N/A** – not analyzed in study, **No change** – no significant effect of exercise

**↑** indicates significant increase in response to intervention, **↓** indicates significant decrease in response to intervention

AA: Amino Acids; BCAA: Branched-Chain Amino Acids; GABA: Gamma-Aminobutyric Acid; HRR: Heart Rate Reserve; HIIT: High-Intensity Interval Training; IBD: Inflammatory Bowel Disease; MET: Metabolic Equivalent of Task; PAG: Physical Activity Guidelines; RM: Repetition Maximum; RPE: Rating of Perceived Exertion; SCFA: Short-Chain Fatty Acids; TMAO: Trimethylamine N-Oxide; VO2 max: Maximum Oxygen Consumption
