# Supplementary material for: A systematic review on the effects of exercise on gut microbial diversity, taxonomic composition, and microbial metabolites: identifying research gaps and future directions
Source: Front Physiol. 2023 Dec 19;14:1292673. doi: 10.3389/fphys.2023.1292673 (PMC10770260; doi:10.3389/fphys.2023.1292673)
Supplement: Supplementary file 4 [file Table4.docx]

**Supplementary Table 4**. Characteristics of the studies included in the systematic review examining the effects of exercise intervention on gut microbiome outcomes in animal models.

| Study | Design | Total no. of subjects | Intervention | Duration | Animal model profile | Phylum | Genus | Diversity | Gut metabolites |
| --- | --- | --- | --- | --- | --- | --- | --- | --- | --- |
| Castro et al. 2021 | Randomized (2 groups) | 10 | Exercise (n=5): resistance training 3x/week on vertical ladder with 4 series of progressive loads @ 50%, 75%, 90%, 100% of MCC (75% of body weight); on completion of 100% load, 30 g added until failure  Control (n=5): sedentary | 12 wk | Male Wistar Rats, age: 45 days, mean body weight: 216.5±10.9 g; 2-3 animals/cage | N/A | Exercise vs. control: ↓ Pseudomonas ↓ Serratia ↓ Comamonas ↑ Coprococcus_1 | Exercise vs. control: ↑ Chao1 index after 12 wk   Exercise vs. control: Altered unweighted unifrac after 12 wk | N/A |
| Chen et al. 2021 | Randomized (4 groups) | 20 | Control: unrestricted access to run   20-minute exercise: 20 minutes on wheel fatigue tester  40-minute exercise: 40 minutes on wheel fatigue tester  60-minute exercise: 60 minutes on wheel fatigue tester | 4 wk | Female C57BL/6 WT mice, age: 4-5 wk | Exercise over 4 wk: ↑ Bacteroidetes ↓ Firmicutes ↑ Verrucomicrobia | Exercise vs. control at 4 wk: ↑Akkermansia, Clostridium, Parabacteroides, Christensenella, Dorea, Roseburia, and Paraprevotella  ↓ Anaerotruncus, Jeotgalicoccus, Flexispira, Alistipes, Ruminococcus, and Desulfovibrio | Exercise vs. control at 4 wk: ↑ Observed OTUs ↑ Shannon's index ↓ Simpson's index  Different Bray–Curtis dissimilarity | Exercise vs. control at 4 wk: ↓ serum D-Lac, LPS, and DAO |
| De Almeida et al. 2016 | 4 groups | 12 | Exercise (n=3): aerobic training using treadmill and automatic walker alternately 6 days/wk  Car (n=3): Exercise + 10 g/day of L-carnitine powder  Chr (n=3): Exercise + 10 mg/day of chelated chromium  Control (n=3): no exercise or supplementation | 6 wk | Mangalarga Marchador horses; fillies; 2.5 to 3 yr, mean body mass: 330 ± 30 kg | No changes | No changes | Exercise groups vs. control: Jaccard index differences at end of 6 wk | N/A |
| Giacco et al. 2020 | 4 groups | 20 | Chow-fed controls (n=4)  Chow-fed + exercise (n=6): exercise - 5 treadmill runs @ low‐intensity; 2x/day first 2 days, and 1 day on 3rd day for 30 min at 15 m/min with 0° incline  Food withdrawal (n=4): food withdrawn for 66 h  Food withdrawal + exercise (n=6) | 3 days | Male wistar rats; age: 13 wk, mean body weight: 300g | No exercise effects | No exercise effects | No exercise effects | N/A |
| Houghton et al. 2018 | Randomization (2 groups). Total 3 groups. | 19 | PolgA mut/mut sedentary (n=5): no exercise training  PolgA mut/mut exercise (n=7): progressive increase in treadmill training for 2 mon from 17cm/s for 10 min to 20 cm/s for 40 min/session, 4x/week.  PolgA+/+ sedentary (n=7): no exercise training | 30 wk | Wild-type PolgA+/+; PolgA mut/mut mice; mean body mass 29.7 g; age: 17 wk | PolgA mut/mut exercise and sedentary over 30 wk: ↑ Firmicutes:Bacteriodetes ratio | PolgA mut/mut exercise over 30 wk: ↑ Bacteroides  PolgA mut/mut exercise vs. sedentary at 30 wk post training: ↑ Mucispirillum ↑ Desulfovibrio | PolgA mut/mut exercise vs. sedentary: Differences in Bray-Curtis dissimilarity | N/A |
| Janabi et al. 2017 | Crossover (2 groups) | 8 | Graded exercise test (GXT, n=4): Incremental high-speed running on treadmill at a fixed 6% grade; initial speed of 4 m/s for 1 min and increased increments of 1 m/s every 60 s until fatigue.  Parallel standing control (SC, n=4): horses standing in stalls | Acute | Unfit, healthy, Standardbred horses, 4 mares and 4 geldings, 3-8 yr, approx. 500 kg | No changes | SC ↓ Clostridium (P=0.03)   No exercise effects. | SC ↓ Shannon's index at species level  At both the genus and species levels the principle coordinate analysis (PCoA) showed significant separation when the samples collected before SC were compared to those collected after SC | N/A |
| Janabis et al. 2016 | 2 groups | 12 | Exercise (n=8): progressive training on 4 days/wk motorised freestall exercise machine and 1 day/wk on treadmill    Parallel seasonal control (n=4): horses housed in adjacent paddocks | 12 wk | Unfit, healthy, Standardbred horses, 4 mares and 4 geldings, 3-8 yr, 446-517 kg | Exercise: Bacteroidetes ↑ from wk 0 to wk 2 and ↓ from wk 4 to wk 6.  Proteobacteria ↑ from wk 4 to wk 6 to wk 8.  Spirochaetes ↑ from wk 2 to wk 4, and ↓ from wk 8 to wk 10. | Exercise: Clostridium ↑ from wk 0 to wk 2 and ↓ wk 2 to wk 4.  Dysgonomonas ↑ from wk 2 to wk 4 and ↓ from wk 4 to wk 6 | Exercise: Shannon diversity (phyla) ↓ from wk 0 to wk 2 and ↑ from wk 4 to wk 6 | N/A |
| Lamoureux et al. 2017 | 4 groups | 42 | Voluntary exercise (VE, n=10): 24-h access to wheel running for 8 wk  Voluntary non-exercise control (VC, n=10): sedentary  Forced exercise (FE, n=11): forced treadmill running 5x/wk for 40 min @ 15m/min-20m/min for 6 wk  Forced non-exercise control (FC, n=10): placed in empty cage for same duration as FE mice | 6-8 wk | Male and female C57BL/6 mice; age: 6-10 wk | N/A | N/A | No changes | N/A |
| Meng et al. 2020 | Randomized (8 groups) | 40 | Control (n=5): room temperature (24°C–26°C ); no exercise  Exercise alone (n=5): room temperature; treadmill exercise at speed of 25 m/min and slope of 0° for 1 h on alternate days; two 30 min exercise bouts  Acute cold alone(n=5): room temperature; no exercise; cold exposure (3°C–4°C) 4 h before sampling; no exercise  Acute cold + Exercise (n=5)  Intermittent cold (n=5): daily cold exposure for 4 h  Intermittent cold alone (n=5): Cold exposure (3°C–4°C); no exercise  Intermittent cold + exercise (n=5): Daily cold exposure for 4 h  Sustained cold alone (n=5): Cold exposure (3°C–4°C); no exercise  Sustained cold + exercise (n=5) | 5 wk | Sprague Dawley rats with obesity, age: 16 wk; on a high-fat diet | Exercise + sustained cold vs. sustained cold alone: ↑ Proteobacteria (P<0.1) ↓ Cyanobacteria  Exercise + intermittent cold vs. intermittent cold alone: ↑ Proteobacteria ↓ Firmicutes | Exercise only vs. control: ↑ Parabacteroides, Ruminiclostridium 9, Allobaculum, Faecalibaculum, Faecalitalea, Holdemania, Gelria over 5 wk  Exercise + cold exposure vs. cold exposure alone: ↑ Prevotella9, Psychrobacter, Oligella, Jeotgalicoccus, RuminococcaceaeTCG-004, [Eubacterium]halliigroup, Facklamia, Paenalcaligenes, Holdemania, Paracoccus, Sporosarcina, CandidatusSoleaferrea over 5 wk | Exercise only: ↑ Shannon’s index over 5 wk  Exercise + acute cold: Shifts in bray-curtis, and unweighted unifrac over 5 wk  Exercise + sustained cold: Shifts in bray-curtis, weighted unifrac, and unweighted unifrac over 5 wk ↓ Shannon’s index over 5 wk  Exercise alone vs. control: Alterations in bray-curtis, weighted unifrac at 5 wk  Exercise + acute cold vs. acute cold only: Alterations in bray-curtis, unweighted unifrac at 5 wk | N/A |
| Mika et al. 2015 | Randomized (2 groups) | 20 | Exercise (n=10): voluntary wheel running  Control (n=10): sedentary in standard cages | 6 wk exercise + 25 days after running stopped | Male adult Fischer F344 rats, age: 10 wk | No changes | Exercise over 6 wk:  ↑Rikenellaceae family genera AF12 and an unclassified genus ↑ Turicibacter | No changes | N/A |
| Ribeiro et al. 2019 | Randomized (4 groups) | 40 (only 12 in microbiome analyses) | Standard diet (SD-C, n=10): Standard diet (68.8% carbohydrate, 18.8% protein and 12.4% fat) with no training  Standard diet + training, (SD-T, n=10): SD + training on treadmill running at 50% VO2 max, 5x/week, 30 min/session  High fat diet (HFD-C, n=10): High fat diet (21.3% carbohydrate, 18.4% protein and 60.3% fat) with no training   High fat diet + training (HFD-T, n=10): HFD + training on treadmill running at 50% VO2 max, 5x/week, 30 min/session | 27 wk total (16 wk diet only + 3 wk adaptation period + 8 wk training | Male C57BL/6 mice; age: 4 wk | N/A | SD-T vs. SD-C and HFD-T vs. HFD-C after diet/training period:  ↑Vagococcus ↓Proteus | No training effects. | N/A |
| Walshe et al. 2021 | Randomized (2 groups) | 14 | Treatment (n=7): weight loss of 1%/wk; 5-day weekly walk–trot exercise using automated horse walker; 25-40 min/session with increase from wk 1 to wk 3.  Control (n=7): weight maintenance diet; exercised daily at walk to mimic foraging conditions; total walking time matched treatment group | 6 wk | Male and female horses, mixed breeds (Standardbred, Cob, Cob pony, and pony), age: 4-17 yr, overweight or obese (BCS>6/9) | No changes | Treatment vs. control: ↑ Coprococcus at wk 6 | Treatment:  ↑ Fisher alpha index, richness index, and Simpson index over 6 wk | Treatment vs. control:  No differences in fecal metabolomics profiles at any time point |
| Yang et al. 2021 | Randomized (2 groups) | 30 | Exercise (n=15): treadmill running 5x/week, 60 min/day, slope of 0, @40-59% of HRR  Control (n=15): no exercise | 14 wk | Male C57BL/6 mice; pathogen-free; age: 5 wk; body mass: 18 ± 2 g | Exercise vs. control at 14 wk: ↑ Actinobacteria | Exercise vs. control at 14 wk: ↓Bacteroides ↓Parabacteroides ↓Odoribacter  Exercise over 14 wk: ↑ Bifidobacteria ↑ Coprococcus  ↑ Clostridiales order genus | Exercise vs. control at 14 wk: ↓ Chao1 Index | N/A |

Results from animal studies examining the effects of exercise non the gut microbiome

**N/A** – not analyzed in study, **No change** – no significant effect of exercise

**↑** indicates significant increase in response to intervention, **↓** indicates significant decrease in response to intervention

D-Lac: D-Lactate; DAO: Diamine Oxidase; LPS: Lipopolysaccharide; OTU: Operational Taxonomic Unit; VLT: Velocity Related to Lactate Threshold; WT: Wild Type
